# Potential of Sorghum Polyphenols to Prevent and Treat Alzheimer’s Disease: A Review Article

**DOI:** 10.3389/fnagi.2021.729949

**Published:** 2021-10-06

**Authors:** Nasim Rezaee, W.M.A.D. Binosha Fernando, Eugene Hone, Hamid R. Sohrabi, Stuart K. Johnson, Stuart Gunzburg, Ralph N. Martins

**Affiliations:** ^1^Centre of Excellence for Alzheimer’s Disease Research & Care, School of Medical and Health Sciences, Edith Cowan University, Joondalup, WA, Australia; ^2^Department of Biomedical Sciences, Macquarie University, Sydney, NSW, Australia; ^3^Centre for Healthy Ageing, Health Future Institute, Murdoch University, Murdoch, WA, Australia; ^4^School of Molecular and Life Sciences, Faculty of Science and Engineering, Curtin University, Perth, WA, Australia; ^5^Ingredients by Design Pty Ltd., Lesmurdie, WA, Australia; ^6^CWEK Pty Ltd., Perth, WA, Australia

**Keywords:** Alzheimer’s disease, sorghum, polyphenols, antioxidant, amyloid-beta, tau, mitochondrial dysfunction, flavonoids

## Abstract

Alzheimer’s disease (AD) is characterized by the excessive deposition of extracellular amyloid-beta peptide (Aβ) and the build-up of intracellular neurofibrillary tangles containing hyperphosphorylated tau proteins. This leads to neuronal damage, cell death and consequently results in memory and learning impairments leading to dementia. Although the exact cause of AD is not yet clear, numerous studies indicate that oxidative stress, inflammation, and mitochondrial dysfunction significantly contribute to its onset and progression. There is no effective therapeutic approach to stop the progression of AD and its associated symptoms. Thus, early intervention, preferably, pre-clinically when the brain is not significantly affected, is a better option for effective treatment. Natural polyphenols (PP) target multiple AD-related pathways such as protecting the brain from Aβ and tau neurotoxicity, ameliorating oxidative damage and mitochondrial dysfunction. Among natural products, the cereal crop sorghum has some unique features. It is one of the major global grain crops but in the developed world, it is primarily used as feed for farm animals. A broad range of PP, including phenolic acids, flavonoids, and condensed tannins are present in sorghum grain including some classes such as proanthocyanidins that are rarely found in others plants. Pigmented varieties of sorghum have the highest polyphenolic content and antioxidant activity which potentially makes their consumption beneficial for human health through different pathways such as oxidative stress reduction and thus the prevention and treatment of neurodegenerative diseases. This review summarizes the potential of sorghum PP to beneficially affect the neuropathology of AD.

## Introduction

Alzheimer’s disease (AD) is a progressive neurodegenerative disease characterized by different neuropathological features including excessive accumulation of Aβ peptides outside of neurons and the hyperphosphorylated form of tau protein inside neurons (Duyckaerts et al., 2009). Currently, more than 50 million people worldwide and more than 440,000 Australians are living with AD (Dementia Australia, 2018a; WHO, 2021). The number of affected people worldwide is expected to reach 152 million by 2050. According to the Australian Bureau of Statistics in 2017, AD was the first leading cause of mortality in Australian women and second leading cause of mortality in all Australians (Australian Bureau of Statistics, 2017). The estimated cost of dementia in Australia was more than $15 billion in 2018 and it will be more than $18.7 billion by 2025, and more than $36.8 billion by 2050 (The National Centre for Social and Economic Modelling NATSEM, 2016).

Despite advances in medicine and drug therapies, a disease-modifying treatment of AD is still not available. Recently, the new drug (Aduhelm) has been approved by the FDA as a modulator of amyloid plaques in the brain but its efficacy on memory and cognition is yet to be determined (Alexander et al., 2021; Canady, 2021). Current drug interventions only temporarily delay the progression of some of the cognitive symptoms of AD. Acetylcholine is a major neurotransmitter in the brain which has limited reserves in the AD brain where the enzymes cholinesterase breaks down the beneficial acetylcholine. Thus, cholinesterase inhibitors including acetylcholinesterase and butyrylcholinesterase inhibitors (AChEI and BChEI) help to attenuate the AD symptoms. The U.S. Food and Drug Administration (FDA) has approved five symptomatic drugs for the treatment of AD, including three cholinesterase inhibitors (rivastigmine, galantamine, donepezil) and memantine and memantine mixed with donepezil (Alzheimer’s Association, 2019). These treatments reduce the rate of progression of cognitive decline by increasing the neurotransmitters in the brain, but they are unable to prevent irreversible damage to neurons (Sivaraman et al., 2019). Therefore, there is a serious need to find a reliable intervention that can prevent or slow AD progression.

Natural sources of antioxidants have been identified as a promising preventive or therapeutic avenue for neuroprotection. Polyphenols (PP) are the most abundant antioxidants in the diet (Kulshreshtha and Piplani, 2016). Diets rich in PP are considered neuroprotective due to their capacity to affect several cellular pathways, that contribute to the pathogenesis of AD, will be discussed below (Malar and Devi, 2014; Lakey-Beitia et al., 2015; Omar et al., 2017).

Sorghum grain is a natural source of antioxidants with strong anti-inflammatory activities (Burdette et al., 2010; Xiong et al., 2019). The profile of PP of sorghum is unique and epidemiological evidence have demonstrated these PP may have specific health benefits such as superior chemoprotective properties and strong anti-inflammatory activity which are not provided by PP in other grains, such as rice, oats, and wheat (Awika, 2011). In addition, several PP of sorghum are thought to beneficially interfere with pathological changes in AD, such as Aβ and tau accumulation in *in vitro* and *in vivo* level (Rossi et al., 2008; Jabir et al., 2018) However, research is yet to identify the effect of sorghum PP on AD pathology.

This review will focus on the current evidence and potential mechanisms for protective effects of sorghum PP on the pathology of AD.

## Alzheimer’s Disease and Associated Pathological Hallmarks

Dementia is a group of disorders characterized by progressive cognitive impairment which affects daily living activities (Roman, 2002). Dementia is considered as one of the most serious health and social concerns of the century. It has major impacts on individuals, carers, families, and societies.

**Figure 1:**
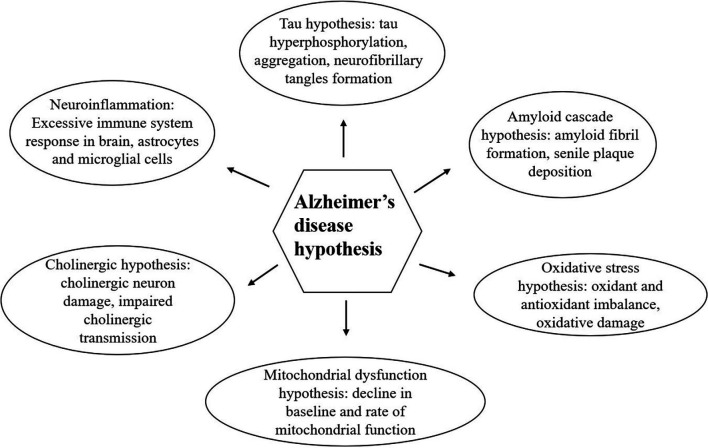
Alzheimer’s disease (AD) hypotheses (Swerdlow, 2018; Altinoglu and Adali, 2020). The six most common AD hypotheses include the amyloid cascade hypothesis, tau hypothesis, neuroinflammation, cholinergic hypothesis, mitochondrial dysfunction hypothesis, and oxidative stress hypothesis which are explained separately through the article. All the these mechanisms can interact with each other.

Alzheimer’s disease is the most prevalent form of dementia (Puglielli et al., 2003) with the clinical symptoms of progressive memory decline and other cognitive functions, eventually leading to an inability to do daily tasks and a reliance on care (Long and Holtzman, 2019). It is pathologically characterized by the accumulation of extracellular Aβ oligomers, hyper-phosphorylation of intracellular neurofibrillary tangles (NFTs; tau protein) and neuroinflammation in the brain (Sadhukhan et al., 2018). Other primary changes of AD include increased oxidative stress, mitochondrial dysfunction, and neuroinflammation (Mecocci et al., 2018). A diagram of the generally accepted hypothesis for AD is shown in Figure 1.

### Aβ Accumulation

**Figure 2:**
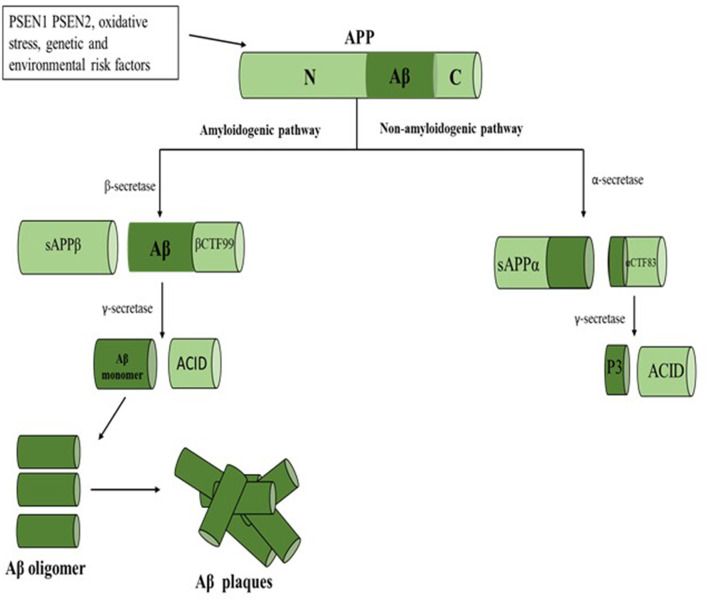
Schematic of Aβ hypothesis. APP is sequentially cleaved *via* amyloidogenic or non-amyloidogenic pathways. In the non-amyloidogenic pathway, it is cleaved by α-secretase, resulting in the production of the sAPPα. In the amyloidogenic pathway, APP is initially cleaved by β-secretase, then γ-secretase, resulting in the production of Aβ peptides. Abbreviations: APP, amyloid precursor protein; *PSEN1*, presenilin 1; *PSEN2*, presenilin 2; Aβ, amyloid beta.

According to the amyloid hypothesis (Hardy and Higgins, 1992), the primary cause of AD is the accumulation and deposition of oligomeric or fibrillar Aβ peptides. The Aβ peptide consists of 38–42 amino acids that are derived from amyloid precursor protein (APP); a transmembrane protein that has two competing pathways. In the non-amyloidogenic pathway, it is cleaved by α-secretase, to produce the secretory fragment sAPPα (see Figure 2). The candidate enzymes for α-secretase which are from the a-disintegrin and metalloprotease (ADAM) family include ADAM17, ADAM9, and ADAM10 (Buxbaum et al., 1998; Lammich et al., 1999; Fahrenholz et al., 2000; Asai et al., 2003). Among these enzymes, ADAM10 is suggested as the most physiologically relevant α-secretase in neurons (Anders et al., 2001; Kuhn et al., 2010). In the amyloidogenic pathway, APP is initially cleaved by β-secretase (BACE1), then γ-secretase, resulting in the generation of Aβ peptides (Gandy et al., 1994). The non-amyloidogenic pathway is beneficial since sAPPα has neuroprotective activity (Corrigan et al., 2011). In contrast, in the amyloidogenic pathway, an over-production of Aβ and its accumulation results in cytotoxicity (Chasseigneaux and Allinquant, 2012; Paroni et al., 2019). The length of the Aβ peptide influences this toxicity, where Aβ_42_ (42 amino acids) is more cytotoxic than Aβ_40_ and Aβ_43_ (Fu et al., 2017). This is because Aβ_42_ has a higher hydrophobicity and thus higher propensity to aggregate by hydrophobic bonding into toxic oligomers compared with Aβ_40_ peptide (Vion et al., 2018). Several inherited and environmental factors such as APP, presenilin 1 (*PSEN1*), and presenilin 2 (*PSEN2*), gene mutations, deficit Aβ clearance, oxidative stress and mitochondrial dysfunction might be contributing factors to the over-production and accumulation of Aβ (Mao and Reddy, 2011; Hernández-Zimbrón and Rivas-Arancibia, 2015; Zuo et al., 2015; Paroni et al., 2019). Although amyloid deposition is always seen in AD patients, its pathogenic role is still unclear (Modrego and Lobo, 2019). While many questions still remain unanswered regarding the pathogenesis of AD, the amyloid hypothesis is still the most accepted theory to describe the associated neuropathological events.

### Tau Proteins

**Figure 3:**
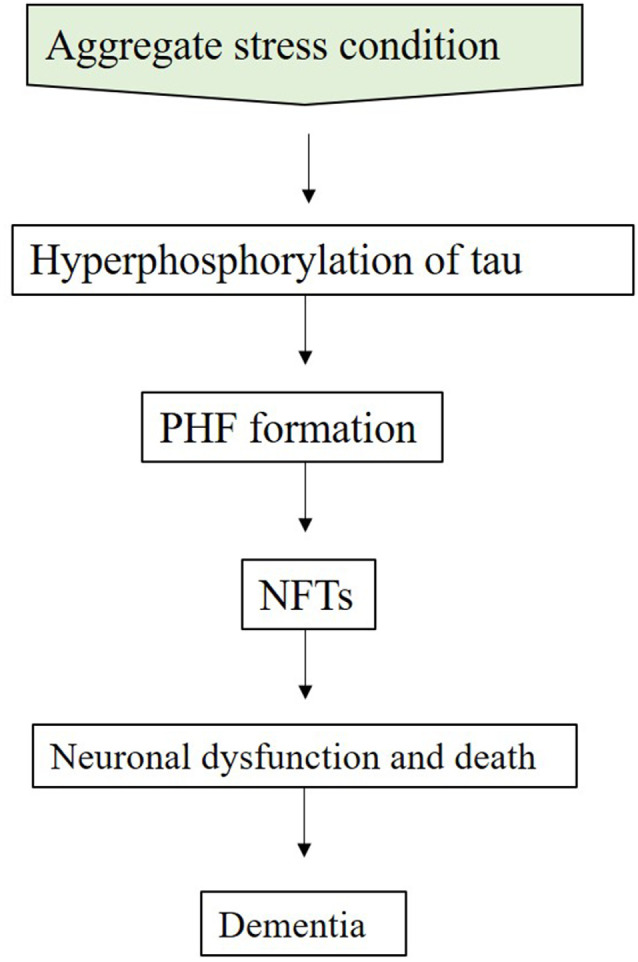
Schematic of tau hypothesis. Aggregate stress condition is a condition in which Aβ aggregation alters the kinase/phosphatase activity that can lead to hyperphosphorylation of tau resulting in PHF formation which consequently leads to neuronal dysfunction and dementia (Verwilst et al., 2018). Abbreviations: PHF, paired helical filament; NFT, neurofibrillary tangles.

Tau proteins are phosphoproteins present in all cells of the central nervous system (CNS; Lionnet et al., 2018). The main function of tau is the modulation of microtubule stability which forms the main pathway for intracellular protein trafficking (Mandelkow, 1998; Buée et al., 2000). But in AD, abnormal hyperphosphorylation of tau leads to its dysfunction, resulting in impairment of the transport system, the cytoskeleton, intracellular signaling, and mitochondrial integrity (Mandelkow, 1998; Iqbal et al., 2005). Hyperphosphorylated tau proteins dissociate from the microtubule (Figure 3) and bind with each other, forming paired helical filaments (PHFs). These accumulate, resulting in the characteristic NFTs seen in AD pathology (Gamblin et al., 2003).

There are other alternative hypotheses where Aβ plaques and NFTs may be formed independently and may be the products of dementia and not the cause (Hardy and Selkoe, 2002; Selkoe and Hardy, 2016). To date, there is no satisfactory hypothesis that can fully explain the exact mechanism of Aβ and tau accumulation, aggregation, and subsequent toxicity.

### Neuroinflammation

The neuroinflammation is a response of the innate immune system within the brain as shown by an increased level of activated microglia and astrocytes, activated complement proteins and cytokines (Heneka et al., 2015; Zhang and Jiang, 2015). In AD, Aβ plaques and NFTs exacerbate any chronic inflammatory state, resulting in the increased action of cytokines (interleukin 1, tumor necrosis factor), prostaglandins, growth factors, thromboxanes, and ROS. These, in turn, enhance the APP processing, increasing Aβ_42_ levels in brain (Meraz-Ríos et al., 2013). Aβ also activates proinflammatory cytokines and some pro-inflammatory enzymes, such as cyclooxygenase-2 (COX-2), inducible nitric oxide synthase (iNOS) and nuclear factor kappa B (NF-κB). This Aβ-linked inflammatory response has been claimed to lead the neuronal damage in AD (Meraz-Ríos et al., 2013; Heneka et al., 2015; Zhang and Jiang, 2015). Accumulating evidence suggests that neuroinflammation is a major contributor of AD onset and progression (Heneka et al., 2010). Long-term consumption of nonsteroidal anti-inflammatory drugs (NSAIDs) can delay the onset or progression of AD, which also supports the role of neuroinflammation in AD (Ali et al., 2019).

### Oxidative Stress as Another Major Contributor to AD Pathology

A growing body of literature indicates that oxidative stress is another pathophysiological feature of the AD brain (Good et al., 1996; Agostinho et al., 2010). Oxidative stress is the imbalance between the formation and detoxification of reactive oxygen species (ROS; Adwas et al., 2019). These ROS are normally produced as by-products of oxygen metabolism which utilizes both free radicals and non-free radical oxygen intermediate species, such as hydrogen peroxide (H_2_O_2_), superoxide ($O_{\text{2}}^{-}$), hydroxyl radical (^•^OH), and singlet oxygen (^1^O_2_). These ROS are known to damage many biomolecules including DNA, RNA, protein, and lipids (Pham-Huy et al., 2008). Studies suggest that oxidative stress could: (a) be a consequence of Aβ deposition; (b) induce the production of Aβ; (c) be a combination of both: (a) and (b) (Sonnen et al., 2008; Tamagno et al., 2012). ROS are produced *in vivo* during oxidation and are involved in the progression of various health problems including cellular aging, mutagenesis, cardiovascular problems, diabetes, and neurodegeneration (Halliwell and Gutteridge, 1999; Moskovitz et al., 2002).

A high intake of foods rich in antioxidants may be beneficial to attenuate the ROS-associated problemsbased on the result of human dietary intervention studies (Lobo et al., 2010; Liu et al., 2018).

### Mitochondrial Dysfunction as One of the Suggested Contributors of AD Pathology

Mitochondria are responsible for energy homeostasis in cells. Their dysfunction may contribute to the progression of several diseases including cancer, cardiovascular diseases, diabetes and neurodegenerative diseases (de Moura et al., 2010; Wen et al., 2016). A large body of research indicates that dysfunctional mitochondria play an important role in the pathogenesis of AD (Bhatti et al., 2017).

The initial level of mitochondria functions as well as its rate of decline influences AD onset and progression (Swerdlow et al., 2014). When mitochondrial function falls below a critical threshold, abnormal tau phosphorylation processes, amyloid plaque generation, synaptic degeneration, and oxidative stress can result (Lezi and Swerdlow, 2012). Several essential mitochondrial functions such as biogenesis, fission/fusion, and bioenergetics are also associated with AD. This makes mitochondrial dysfunction an important factor to consider in AD pathogenesis and its prevention (Lezi and Swerdlow, 2012; Nicolson, 2014; Flannery and Trushina, 2019).

## Risk Factors for Alzheimer’s Disease

Risk factors are the specific conditions of an individual’s lifestyle along with genetic, gender, and environmental factors that determine the likelihood of developing AD (Alzheimer’s Association, 2018). The risk factors of AD can be divided into two main groups. Modifiable risks, are those that can be reduced through specific actions such as lifestyle changes. These risks contribute to 40% of AD cases (Livingston et al., 2020). Non-modifiable risks are those that cannot be changed including parental dementia, genetic risk factors and etc. (Alzheimer’s Association, 2018).

### Modifiable Risk Factors

There is compelling evidence that smoking, high blood pressure, diabetes, high cholesterol, and obesity significantly increase the risk of AD (Prince et al., 2014). Hormones (testosterone and estrogen) can have a neuroprotective effect through regulating Aβ, thus, age-related decline in these hormones can affect cognitive ability and therefore increase the risk of developing AD (Verdile et al., 2014). Traumatic brain injury (TBI) is also reported to increase AD risk (Alzheimer’s Association, 2018).

Lifestyle factors including a healthy diet; adequate physical exercise, good sleep hygiene and cognitive training have been shown to reduce the risk of developing AD (Bauer and Morley, 2018). Conversely psychological factors (e.g., depression, anxiety, and stress) and vascular risk factors contribute to an increased risk of AD. A diet with high PP and high antioxidant activity can thus be considered as an approach to assist the prevention of chronic diseases, especially AD.

### Non-modifiable Factors

Age is the main risk factor for AD. As people age, the risk of AD increases exponentially, as shown in several population based studies (Corrada et al., 2010). Apolipoprotein E *(APOE)* ε4 allele is the major genetic risk factor, which increases the probability of developing AD (Thakur et al., 2019). The effects of *APOE* ε4 on cognitive ability are variable from person to person (Prince et al., 2014; O’Donoghue et al., 2018). The *APOE* gene is present in chromosome 19 (Dementia Australia, 2018b). In humans, there are three common alleles: ε2, 3 and 4. Each individual carries two apolipoprotein genes which can be the same type (ε2, 2; 3, 3 or 4, 4), or a combination of two types (ε2, 3; 2, 4; 3, 4; Dementia Australia, 2018b). Individuals with at least one ε4 have a 2 to 3-fold risk of AD while those with two ε4 alleles (4, 4) rarely escape the disease. Compared to the other APOE alleles, the higher risk of developing AD in ε4 alleles is associated with an earlier age of AD onset (Alzheimer’s Association, 2018). This higher risk is three fold for one copy of ε4 allele and 12 fold for two copies (Alzheimer’s Association, 2018). In contrast, *APOE ε2* carriage has a neuroprotective effect relative to *APOE ε3* and *APOE ε4*. Carrying the double-barrelled *APOE* ε*4* combination is fortunately uncommon, affecting only about 2% of the population, whereas about 25% of people carry a single copy of *APOE ε4* (Alzheimer’s Association, 2018).

## Natural Products to Effectively Combat Alzheimer’s Disease

Nutraceuticals (“nutrition” + “pharmaceutical”) with diverse compositions of plant secondary metabolites may hold great potential forpreventing and treating chronic diseases such as AD. These secondary metabolites, below a toxic dosage, usually do not have the side effects seen in synthetic drugs and are more widely available through the agri-food system. Some plant secondary metabolites such as PP perform beneficial physiological acts through specific mechanisms such as targeting enzymes and receptors. Epidemiological and preclinical studies have shown the protective effect of nutraceuticals such as fatty acids and polyphenolics (PP) found in fruits, vegetables, herbs, and nuts against neurodegeneration, to improve memory and cognitive function (Cole et al., 2005; Miller et al., 2017). In light of the literature, dietary PP, one of the richest sources of antioxidant activity in the human diet have become a topic of great current interest as potential neuromodulator agents to attenuate pathological hallmarks of AD. The rationale for this is their potential protective activities such as blood-brain-barrier (BBB) penetration capacity, oxidative stress attenuation, and Aβ aggregation inhibition (Mendes et al., 2018). Taken together, these potential therapeutic effects of PP indicate great potential of this class of phytochemicals to be investigated as a protective agent for AD (Panza et al., 2018).

### Polyphenols and Their Anti-Alzheimer’s Disease Potential

Polyphenols are naturally occurring compounds and secondary metabolites of plants mostly produced in response to major stress (Pandey and Rizvi, 2009; Isah, 2019). They protect plants against biotic (living beings present in an ecosystem e.g., fungi, bacteria, and protists), and abiotic (non-living components e.g., water, soil, air, sunlight, temperature, and minerals) stressors (Rauf et al., 2019) acting as antioxidants, antimicrobials, and photo-absorption molecules. Thus, they defend plants from pathogens, ultraviolet radiation damage and predators such as insect pests (Beckman, 2000). Moreover, they are involved in the structural strength of plants during growth (Pandey and Rizvi, 2009). Polyphenols have received special attention from researchers due to their antioxidant activities which enable them to scavenge free radicals formed during the pathological processes of diseases such as cancer, cardiovascular diseases, and neurodegenerative disorders (Lakey-Beitia et al., 2015). They also have anti-inflammatory activity that is important in reducing oxidative stress thus conferring potential protective effects against the neurodegenerative process (Masci et al., 2015).

Polyphenols have demonstrated that they provide their neuroprotection through antioxidant, cholinergic, Aβ, and tau aggregation pathways *in vitro* and *in vivo* (Omar et al., 2017). The PP attenuate Aβ toxicity and oxidative stress in neurons by decreasing the Aβ aggregation and increasing the scavenging of free radicals, as shown in animal and cell culture studies (Dore et al., 1999; Agostinho et al., 2010; Mathiyazahan et al., 2015; Bai et al., 2017; Hwang et al., 2017). Polyphenols donate electrons to the free radicals to neutralize them, which is important to decrease the levels of ROS within cells (Lobo et al., 2010). Additionally, there is some evidence from cellular and animal model studies that PP may inhibit the Aβ_42_ toxicity (Bastianetto et al., 2008; Hugel and Jackson, 2015). Decrease in the hyperphosphorylation of tau protein, the formation of NFTs, and inflammation in *in vitro* and *in vivo* studies upon addition of PP has also been demonstrated (Mendes et al., 2018).

**Figure 4:**
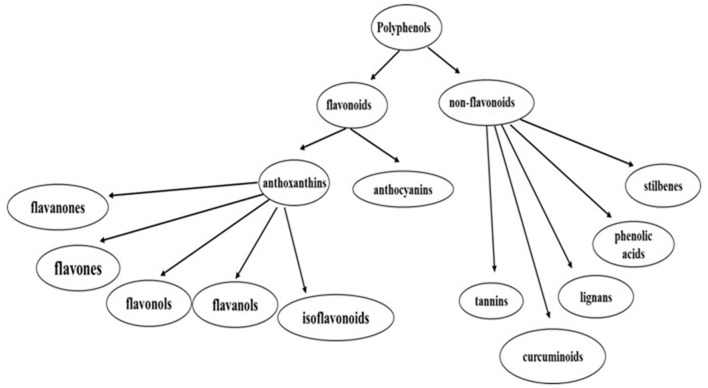
Polyphenols classifications. PP are divided into two main groups of flavonoids and non-flavonoids (El Gharras, 2009; Lakey-Beitia et al., 2015).

The basic structure of PP includes two aromatic rings linked through a pyran ring (Ross and Kasum, 2002). There structures are very complex, with the two main categories of PP are flavonoids and non-flavonoid compounds (El Gharras, 2009; see Figure 4). Flavonoids contain 15 carbon atoms. They are soluble in water and characterized by two benzene rings connected through a three-carbon chain. Flavonoids are sub-divided into anthoxanthins (flavones, flavonols, flavanols, isoflavonoids, flavanones), and anthocyanins (Lakey-Beitia et al., 2015). Non-flavonoid PP are phenolic acids, stilbenes, curcuminoids, lignans, and tannins (Lakey-Beitia et al., 2015).

It has been hypothesized that the, the anti-amyloidogenic activity of PP is due to their physicochemical features, including the aromatic rings, molecular planarity, hydrogen bond formation, internal double bonds, and molecular weights below 500 g/mol (Lakey-Beitia et al., 2015). All these features are important for the inhibition of the amyloidogenic APP processing to reduce amyloid load, by activating α-secretase and inhibiting β- and γ-secretase (Lakey-Beitia et al., 2015).

Several *in vitro* and *in vivo* studies reported that PP-rich extracts from plants, like fruits and herbs, possess neuroprotective activities (Dai et al., 2006; Rossi et al., 2008; Loef and Walach, 2012; Hassaan et al., 2014; Dal-Pan et al., 2017; Omar et al., 2017; Polito et al., 2018). For instance, *in vitro*, *in vivo* and clinical studies showed the neuroprotective action of berry fruits through their polyphenolic contents (Vepsalainen et al., 2013; Wong et al., 2013; Subash et al., 2014). Other *in vitro* and *in vivo* studies indicate that pomegranate which is also rich in PP has the potential to attenuate AD progression by its anti-inflammatory and anti-Aβ accumulation activity (Hartman et al., 2006; Rojanathammanee et al., 2013). Moreover, extracts of other PP-rich fruits such as apple, banana, orange, grape, citrus fruit, and walnut have been also shown to inhibit Aβ neurotoxicity and oxidative stress as demonstrated by several *in vitro* studies (Chauhan et al., 2004; Heo et al., 2008; Toda et al., 2011; Lian et al., 2016; Braidy et al., 2017a). In one animal model study, PP-rich fruits such as Palm fruit could attenuate oxidative stress (Subash et al., 2015). Of particular interest to this current review is that several *in vivo* studies have reported cognition and memory enhancement activities of grapes, citrus fruit, walnut and buckwheat extracts (Wang et al., 2010; Choi et al., 2013; Lian et al., 2016; Braidy et al., 2017a; Pandareesh et al., 2018). *in vitro* investigation of the effect of a PP-rich extract of perennial buckwheat reported attenuation of Aβ toxicity in plasma (Liang et al., 2017). Another *in vitro* study using PP-rich extract of the herb *Patrinina villosa* Juss has shown a significant inhibitory effect on Aβ plaque aggregation (Bai et al., 2017). A cell culture study on twenty different South African medicinal PP-rich plants against AD reported the effectiveness of *Xysmalobium undulatum*, *Cussonia paniculata*, and *Schotia brachypetala* in decreasing the production of Aβ in comparison to other investigated extracts (Thakur et al., 2019). Moreover, based on the dietary intervention animal study of Ingale and Kasture (Ingale and Kasture, 2017), PP rich extract of purple passionflower could enhance cognitive function. Animal model studies, *Capparis spinose*, *Caesalpinia crista*, *Iris germanica*, and *Paeonia suffruticosa* could attenuate inflammation and Aβ aggregation through their polyphenolic contents, and make positive changes in cognition and memory (Costa et al., 2016; Gu et al., 2016; Borhani et al., 2017).

*In vitro* studies have reported that dietary drinks such as the crude juice of broccoli sprouts (Masci et al., 2015), tea (Polito et al., 2018), coffee (Ishida et al., 2018), and red wine (Dhir, 2018) are protective against Aβ-induced cytotoxicity and apoptotic cell death. They have been shown to attenuate mitochondrial dysfunction and hyperphosphorylation of tau proteins through their polyphenolic content (Lakey-Beitia et al., 2015; Sawikr et al., 2017; Polito et al., 2018).

Whole grain consumption as part of a healthy diet has been reported to be protective against several chronic diseases (Miller et al., 2000; Slavin, 2003; Aune et al., 2016). The health benefits of whole grains are in part due with their PP and the associated antioxidant activity (Slavin et al., 1997; Miller et al., 2000; Slavin, 2003; Tian et al., 2019).

Among whole grains, sorghum has some unique features that make it very attractive for neuroprotection studies. It is an inexpensive and abundant grain with a wide range of varieties, some of which are very high in PP content (including PP that are very rarely found in other plant food) and antioxidant activity. Several *in vitro* and *in vivo* studies have reported beneficial effects of sorghum PP on chronic diseases such as diabetes and cardiovascular disease, both of which are as risk factors of AD (Kim and Park, 2012; Suganyadevi et al., 2013; Stefoska-Needham et al., 2015; de Morais Cardoso et al., 2017; Moraes et al., 2018).

## Sorghum

### General Characteristics of Sorghum

**Figure 5:**
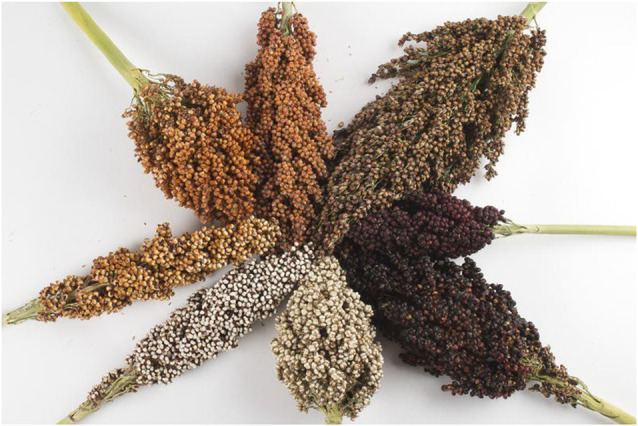
Different genotypes of sorghum grain: black pericarp, red pericarp, white pericarp, brown pericarp, and orange pericarp varieties. Selection of the most potent varieties of sorghum is crucial for health and medical-related purposes. Picture adopted from Barmac (2021).

Sorghum (Figure 5) is the fifth most-produced cereal crop in the world (Awika and Rooney, 2004). It is adaptable to grow in drought and hot climates. Thus, it is usually grown in warm semi-arid and arid areas across the globe (de Morais Cardoso et al., 2017). Sorghum grain has been mostly used as livestock feed and in the biofuel industry (de Morais Cardoso et al., 2017). Sorghum is gluten-free and low-fat while being high in protein and fiber. It has a high antioxidant and anti-inflammatory potential due to its bioactive compounds such as polyphenolics (Awika and Rooney, 2004; de Morais Cardoso et al., 2017).

### Classification of Sorghum

Sorghum grain has been classified into three different groups based on extractable tannin content. Sorghum type I (low tannins extracted by 1% acidified methanol), type II (tannins extractable in 1% acidified methanol and not methanol alone), and type III (tannins extractable in both acidified methanol and methanol alone; Awika and Rooney, 2004).

Another common way of sorghum classification is based on its grain color and its total polyphenols. Sorghum has varieties with pigmented and non-pigmented precarps. White sorghum has no tannins or anthocyanins and has a very low level of total PP. Red sorghum (red pericarp) has a considerable amount of extractable PP without any tannins. Black sorghum (black pericarp) has a large amount of anthocyanins and finally, the brown sorghum (pigmented testa, different degrees of pericarp pigmentation) contains significant levels of tannins (Awika and Rooney, 2004). The concentration of flavonoid in sorghum is related to the pericarp color, pericarp thickness, and presence of testa (Taleon et al., 2012). However both environmental and genetic factors influence the phenolic level and profiles of sorghum grain (Awika and Rooney, 2004).

### Sorghum PP and Health Benefits

A limited numbers of studies such as Awika et al. (Awika and Rooney, 2004; Yang et al., 2009; Awika, 2017; Girard and Awika, 2018) and Cardoso (de Morais Cardoso et al., 2017) have investigated the potential benefit of sorghum on health and disease prevention. According to their findings, sorghum should be considered as a health-beneficial grain, not just a low-value cereal grain. Sorghum has shown a positive impact on glycemic control, colonic microbiota, cholesterol attenuation, cardiovascular disease, anti-mutagenicity, and anti-inflammatory activity (Stefoska-Needham et al., 2015; de Morais Cardoso et al., 2017) which are all risk factors of AD. Below, we summarize information on the effect of sorghum on some chronic disease and their relation to AD.

#### Sorghum Protects Thyroid Gland Function and Combats Obesity

Sorghum is rich in manganese which is essential for thyroid hormone homeostasis. It facilitates the proper regulation of the thyroid gland promoting weight loss through regulating fat metabolism (Kangama, 2017). Moreover, sorghum contains a slow-digestible starch relative to other cereal crops which is also helpful to control obesity through slow glucose release and therefore modulation of food intake (Girard and Awika, 2018; Hasek et al., 2018). These beneficial characteristics of sorghum in controlling thyroid function (van Osch et al., 2004; Tan and Vasan, 2009; Chaker et al., 2016) and obesity (Alford et al., 2018) are considered as important risk factors of AD and thus can be very useful in its prevention.

#### Prevention of Cancer

The sorghum bran layer is rich in antioxidants which may reduce the risk of cancer by eliminating the possibility of free radical damage to DNA (Kangama, 2017). Sorghum extracts have been shown to have an antiproliferative effect on cancer cells (de Morais Cardoso et al., 2017). Suganyadevi et al. (2011) found that the red sorghum anthocyanin has anti-proliferative activity on a breast cancer cell line (Devi et al., 2011). Similar studies have shown the ability of sorghum extractto inhibit of cell proliferation and increase cell cycle regulator leukemia (Woo et al., 2012), breast (Park et al., 2012a), colon (Suganyadevi et al., 2011), and liver (Suganyadevi et al., 2011) cells. This characteristic is potentially beneficial to fight against diseases in which excessive free radicals play a major role including. AD (Kamath et al., 2004). The antioxidant and neuroprotective activity of the red dye extract from sorghum stem on cyclophosphamide-induced oxidative stress in rat brain is attributed its high level of phenolic and antioxidant activities (Oboh et al., 2010).

#### Managing Diabetes

Some varieties of sorghum grain possess a high amount of tannins which interact with starch and inhibits its digestion thus beneficially regulating blood glucose and insulin levels (Kangama, 2017). It has been suggested that sorghum has anti-diabetic and hypoglycemic effects through the regulation of insulin sensitivity *via* peroxisome proliferator-activated receptor gamma (PPAR-γ; Park et al., 2012b). Another study indicated that the hypoglycemic effect of sorghum extract is associated with hepatic gluconeogenesis not the glucose uptake of skeletal muscle (Kim and Park, 2012). Sorghum also can reduce both glucose and insulin responses (Poquette et al., 2014) and promote glucose and insulin homeostasis (Moraes et al., 2018). As diabetes could increase the risk of developing AD and declining cognitive function, anti-diabetic agents such as sorghum PP could potentially attenuate the AD pathological pathways (Arvanitakis et al., 2004; Hölscher, 2011).

#### Anaemia Prevention

The high level of iron and copper in sorghum helps the generation of red blood cells and improves the blood circulation and growth of cells and decreases the probability of getting anemia (Kangama, 2017). According to a population-based study, anemia is also considered as a risk factor of AD in the elderly and therefore preventing anemia would contribute to decreasing the risk of developing AD (Beard et al., 1997).

#### Assisting With Digestion and Cardiovascular Diseases Prevention

Sorghum assists with the proper function of the digestive system through its dietary fiber content (Kangama, 2017). This helps to control bloating, constipation, diarrhea, and excess gas. Moreover, having a high level of fiber in the diet decreases cholesterol uptake binding bile acids in the small intestine and preventing them from entering the blood-stream which is helpful for the prevention of cardiovascular which includes atherosclerosis, and stroke (Knopp et al., 1999; Kangama, 2017). Cardiovascular disease is considered an important risk factor for AD and thus its prevention will help reduce its risk (Meyer et al., 2000; Tosto et al., 2016; Tini et al., 2020).

### The Anti-Alzheimer’s Disease Potential of Sorghum Polyphenolics

Some varieties of sorghum possess up to 6% (w/w dry basis) of phenolic compounds which is the highest level in any cereal grain (Su et al., 2017). Almost all classes of the phenolic compounds are present in sorghum (Awika and Rooney, 2004) including phenolic acids, flavonoids, tannins, and stilbenes (Tables s 1, 2; Vanamala et al., 2017). The bran fraction of sorghum has the highest concentration of PP thus processing to remove the bran (decortication), will notably decrease the potential health benefits of the grain and therefore un-decorticated sorghum (whole grain) is recommended for consumption (Girard and Awika, 2018; Ashley et al., 2019).

**Table 1:** Reported flavonoids in sorghum (Vanamala et al., 2017).

Class	Compound
Proanthocyanidins	(3-Deoxyanthocyanidins)
	Apigeninidin
	Luteolinidin
	7-methoxyapigenindin
	5-methoxyluteolinidin
	malvidin
Flavones	apigenin
	luteolin
	tricin
Flavanones	naringenin
	eriodictyol
	eriodictyol 5-glucoside
Flavonols	kaempferol 3-rutinoside-7-glucuronide
	quercetin 3,4’-dimethyl ether
Dihydroflavonols	taxifolin
	taxifolin 7-glucoside
Flavan-3-ols	catechin
	epicatechin
	procyanidins

**Table 2:** Reported non-flavonoid in sorghum (Vanamala et al., 2017).

Class	Compound
Phenolic acids	Protocatechuic acid, p-hydroxybenzoic acid, vanillic acid, p-Coumaric acid, o-Coumaric acid, Ferulic acid, Gallic acid, gentisic acid, Caffeic acid, Cinnamic acid, Hydroxybenzoic acid, Salicylic acid, Syringic acid, Sinapic acid
Stilbenes	trans-resveratrol, trans-piceid

**Figure 6:**
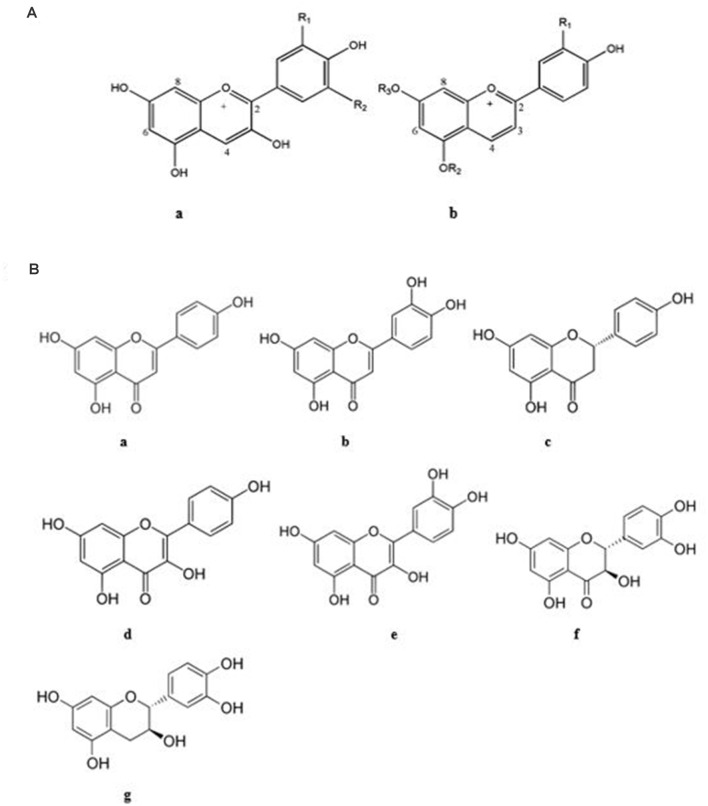
Chemical structure of important flavonoids of sorghum with anti-AD activities. **(A)** Anthocyanidins (**a**: R_1_= R_2_ = OCH_3_: malvidin, R1 = OH, R2 = H: cyanidin, R1=OCH_3_, R_2_ = H: peonidin, **b**: R_1_ = H, R_2_ = H, R_3_= H: apigeninidin, R_1_ = H, R_2_ = Glc, R_3_= H: apigeninidin-5-glucoside, R_1_ = H, R_2_ = H, R_3_=CH_3_: 7- methoxyapigenindin, R_1_ = OH, R_2_ = H, R_3_= H: luteolinidin, R_1_ = OH, R_2_ = Glc, R_3_= H: luteolinidin-5-glucoside, R_1_ = OH, R_2_=CH_3_, R_3_= H: 5-methoxyluteolinidin), **(B)** others (**a**: apigenin **b**: luteolin **c**: naringenin **d**: kaempferol **e**: quercetin **f**: taxifolin **g**: catechin; Awika et al., 2005; Lakey-Beitia et al., 2015; Vanamala et al., 2017; Jabir et al., 2018).

**Figure 7:**
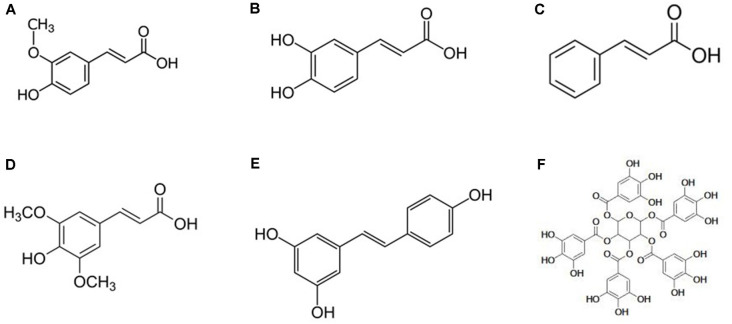
Chemical structure of important non-flavonoids of sorghum with anti-AD activities (Lakey-Beitia et al., 2015; Girard and Awika, 2018; Jabir et al., 2018; **A**: ferulic acid, **B**: caffeic acid, **C**: cinnamic acid, **D**: sinapic acid, **E**: resveratrol, and **F**: tannins).

Based on the literature, almost all the polyphenolic compounds of the various sorghum genotypes have antioxidant activity which may be effective for the attenuation of AD pathological hallmarks (Awika and Rooney, 2004). Among all the PP of sorghum (Tables s 1, 2), caffeic acid, trans-resveratrol, quercetin, catechin, cinnamic acid, cyanidin, apigenin, and kaempferol have gained the most attention for AD prevention and treatment (Rossi et al., 2008; Jabir et al., 2018). Snow et al. (2019) showed that PP exert their anti-AD properties primarily through prevention of aggregation of Aβ fibrils and tau protein NFTs. The presence of hydroxyl groups adjacent to aromatic rings may enhance the inhibition of Aβ/tau aggregation (Snow et al., 2019) by reducing the secondary folding of β-sheet structures which are characteristic of Aβ plaques and NFTs. For example, this property is found in proanthocyanidins, which are highly effective in reducing plaques and tangles in the brain as well as in improving short-term memory. Concluding from this article, sorghum PP such as epicatechin, luteolin, quercetin, etc. with adjacent hydroxyl groups can provide Aβ/tau disaggregation (Figures 6, 7).

Collectively the functions of sorghum PP include a combination of antioxidant, anti-amyloid, anti-tau, anti-inflammatory, AchEI and BChEI activities (Rossi et al., 2008; Omar et al., 2017; Jabir et al., 2018).

In summary, sorghum has a diverse polyphenolic profile depending on its genotype. According to *in vitro* and *in vivo* studies, several PP of sorghum have the potential to act as an anti- AD agent through different pathways such as free-radical scavenging, inhibition of Aβ and tau aggregation. The neuroprotection potential of important single PP of sorghum is illustrated in the subsequent sections.

#### Flavonoids

##### Proanthocyanidins

Pigmented sorghums are rich in anthocyanins some of which are rarely found elsewhere in plants kingdom (Su et al., 2017). High levels of different anthocyanins were detected in a red seed variety PI297139 (1,461.4 ± 98.7 mg/kg), followed by two brown varieties PI221723 and PI35038 (1,376.4 ± 33.2, 937.3 ± 29.4 mg/kg, respectively) and a yellow variety PI229838 (574.8 ± 105.4 mg/kg; Su et al., 2017).

Anthocyanins are divided into the sugar-free proanthocyanidins aglycons and the anthocyanin glycosides (Khoo et al., 2017). The most common anthocyanins found in the plant include cyanidin, delphinidin, pelargonidin, peonidin, malvidin, and petunidin (Khoo et al., 2017). Anthocyanins have several health benefits, where their intake is associated with a lower risk of several chronic diseases due to vasoprotective and anti-inflammatory activities (Lietti and Picci, 1976), antioxidant (Ali et al., 2018), anticancer, chemoprotective (Karaivanova et al., 1990), and hypoglycemic properties (Tsuda et al., 2003). In addition, anthocyanins also are beneficial in the progression/managing/controlling of cardiovascular diseases and HIV-1 (Nakaishi et al., 2000; Stintzing et al., 2002; Cooke et al., 2005; Jang et al., 2005; Julie Beattie and Duthie, 2005; Talavéra et al., 2006).

Anthocyanins are a type of PP with the ability of crossing the blood brain barrier (BBB; Belkacemi and Ramassamy, 2016). It is also reported that anthocyanins from anthocyanin-enriched bilberry and blackcurrant extracts can regulates the APP processing and spatial memory of a mouse model of AD (Vepsäläinen et al., 2013). Additionally, they could alleviate cognitive dysfunction and neuroinflammation in APP/PSEN1 transgenic mice model of AD (Li et al., 2020). According to the cell culture study of Belkacemi and Ramassamy, a mixture of anthocyanins and proanthocyanidins could beneficially affect various mechanisms involved in AD development such as through inhibition of Aβ toxicity and tau phosphorylation, prevention of oxidative stress, and amelioration of mitochondrial dysfunction (Belkacemi and Ramassamy, 2016).

The most common group of anthocyanins found in sorghum are the 3-deoxyanthocyanidins (3-DXA) and their derivatives (Hipskind et al., 1990; Figure 6A). Sorghum is the only common dietary source of the 3-DXA. The percentage of the 3-DXA in sorghum is dependent on the genotype (Awika and Rooney, 2004; Su et al., 2017). The recent animal study of Arbex et al. (2018) suggested that 3-DXA have a significant anti-inflammatory effect, thus protecting against one of the main hallmarks of AD (Arbex et al., 2018). The 3-DXAs are also reported to have antioxidant and anti-carcinogenic activities (Fratianni et al., 2007; Suganyadevi et al., 2011; Makanjuola et al., 2018). However, the data on the bioactivity of 3-DXA are very limited (Dore et al., 1999; Mathiyazahan et al., 2015). Also, there is lack of clinical trial data on the neuroprotective activity of anthocyanins and specifically 3-DXA.

Two main 3-DXAs of the sorghum are luteolinidin and apigeninidin which are orange and yellow colored, respectively. These two 3-DXAs which lack oxygen at the C-3 position are very rare in nature and also differ from the more common proanthocyanidins. The lack of oxygen at C-3 is associated with their high stability in light, heat, and change in pH (Suganyadevi et al., 2013; Figure 6A). Also, the molar absorptivity of the 3-DXA is higher compare to other proanthocyanidins except for cyanidin-3,5-diglucoside and therefore 3-DXA will be absorbed higher than other anthocyanins (Awika et al., 2004).

##### Flavones

Flavones are an important subgroup of flavonoids that are abundant in plants, especially herbs and cereal grains such as maize and sorghum (42–386 μg/g; Jiang et al., 2016). Common flavones include apigenin, luteolin, tangeritin, and chrysin (Singh et al., 2014; Kawser Hossain et al., 2016). Flavones have beneficial functions not only for the plant but also for human health. They possess strong antioxidant activity, which makes them potentially beneficial for the prevention and treatment of several chronic diseases including cancer, coronary heart disease, and neurodegenerative disease (Martens and Mithöfer, 2005; Singh et al., 2014).

###### Apigenin

Apigenin is present in several plants including sorghum with the reported concentration of 0.36–2.09 μg per gram of dry grain (Bradwell et al., 2018). This flavone from sorghum is known to be a strong antioxidant (Makanjuola et al., 2018). It has been shown to protect neurites and cell viability by enhancing the cytokine and nitric oxide release in inflammatory cells which may prevent or slow the progression of AD (Balez et al., 2016). Several studies reported the anti-inflammatory effects of apigenin in human and animal experiments (Liang et al., 1999; Rezai-Zadeh et al., 2008; Zhang et al., 2014). In one study, they induced inflammation by adding lipopolysaccharide to human and mouse macrophages then checked the effect of apigenin by PrimePCR array and through examining different mechanisms. They identified major target genes regulated by apigenin in lipopolysaccharide-mediated immune response (Zhang et al., 2014).

According to the apigenin-treated animal study of Zhao et al. (2013), in a double transgenic mouse model of AD, apigenin ameliorated the APP processing and Aβ toxicity through the regulation of BACE1 level and the reduction of Aβ deposition. They also showed apigenin beneficially reduced oxidative stress and reduced memory impairment, reduced of oxidative stress through the Morris water maze performance test (Zhao et al., 2013). It is also reported that oral administration of apigenin ameliorated the learning and memory deficits of Aβ-induced mice by attenuating oxidative damage, enhancing cholinergic neuronal transmission, and maintaining the BBB integrity in the cerebral cortex (Liu et al., 2011).

###### Luteolin

Luteolin is a flavone class of flavonoids found in several plants including sorghum (Lin et al., 2008). The concentration of luteolin in sorghum grain is (0.84–5.57 μg/g, dry basis) depending on the variety and environmental factors (Bradwell et al., 2018). Luteolin showed strong antioxidant and neuroinflammation activities in *in vitro* and *in vivo* studies (Paterniti et al., 2014; Kwon, 2017). Based on the animal model study of Wang et al. (2016) which was performed through Morris water maze and probe tests, luteolin (10 and 20 mg/kg) significantly attenuates spatial learning deficiencies and memory impairment. Furthermore, the animal behavioral tests study of Yu et al. (2015) found that luteolin (200 mg/kg) attenuates Aβ-induced learning and memory impairment through increasing the regulation of the cholinergic function and attenuation of oxidative stress. According to another *in vivo* study, luteolin could improve basal synaptic transmission and enhance the long-term potentiation (which is considered as a cellular correlate of learning and memory) through high frequency stimulation in the dental gyrus of the rat hippocampus (Xu et al., 2010). Moreover, daily oral administration of luteolin (50, 100, and 200 mg/kg) demonstrated a significant reduction of Aβ aggregation, oxidative stress, and inflammatory reaction in the hypoperfused rat brain (Fu et al., 2014).

##### Flavanones

Flavanones are a subgroup of flavonoids commonly available in grains and especially sorghum and some fruits such a citrus fruit (Tomás-Barberán and Clifford, 2000; Duodu and Awika, 2019). Common flavanones include hesperidin, naringenin, isosakuratenin, and eriodictyol (Das et al., 2019).

Some varieties of sorghum such as yellow sorghum possess high levels of flavanones mainly eriodictyol and naringenin; up to 1,800 μg/g depending on genotypes and environmental factors. The levels of reported flavanones in sorghum grain are much higher than in citrus fruit (400–600 μg/g) which has previously been considered as a main source of these compounds (Duodu and Awika, 2019).

According to cell culture studies, flavanones have several potential health benefits including neuroprotection potential through scavenging ROS (Lu et al., 2010), inhibiting the H_2_O_2_-induced neurotoxicity, increasing catalase activity, attenuating the intracellular free Ca^2+^, and decreasing the mitochondrial membrane potential (Hwang and Yen, 2008).

###### Naringenin

Naringenin is a compound that belongs to the flavanone group of flavonoids. It is available in several plant food including citrus fruits and sorghum (Manchope et al., 2017). Several *in* *vivo* and *in vitro* studies reported anti-inflammatory and antioxidant activity of naringenin (Heo et al., 2004; Manchope et al., 2017). The study of Khajevand-Khazaei et al. (2018) suggested positive effects of naringenin for the alleviation of lipopolysaccharide-induced cognitive deficits in rats; through enhancing the spatial recognition memory in Y maze, discrimination ratio in the object discrimination task, and retention in the passive avoidance test. Lipopolysaccharide and naringenin were administrated daily in a dose of 167 μg/kg and 25, 50, or 100 mg/kg, respectively (Khajevand-Khazaei et al., 2018). Naringenin is able to pass through the BBB and thus can act on the CNS. It has been shown to increase Aβ degrading enzymes through increasing M2 microglia polarization and inhibiting Aβ_42_ -induced M1 microglia activation in primary cultured cortical microglia (Yang et al., 2019). Two other *in vivo* studies also showed the ability of naringenin to improve learning and memory function through alleviation of oxidative stress and reducing apoptosis as evidenced by the Morris water maze test conducted in a rat model of AD (Ma et al., 2013; Ghofrani et al., 2015).

##### Flavonols

Flavonols are another subgroup of flavonoids which commonly available in onions, leeks, broccoli, blueberries and also abundant in cereal such as quinoa, barley, and sorghum (Awika, 2011; Pérez-Chabela and Hernández-Alcántara, 2018).

Common dietary flavonols include myricetin, quercetin, and kaempferol (Aherne and O’Brien, 2002). Flavonols are reported to have several health benefits including inhibiting low-density lipoprotein oxidation and thus reduced risk of atherosclerosis and general cardio protection effects (Giovinazzo and Grieco, 2019), cancer (Ali et al., 2008; Szliszka et al., 2011), and neurogenerative disease such as brain vascular atrophy, mild cognitive impairment (MCI), and AD (Patel et al., 2008).

*Kaempferol* is found in a variety of plants including sorghum (Przybylska-Balcerek et al., 2019). Epidemiological evidence suggests a positive relationship between the high intake of kaempferol and a reduced risk of developing several chronic diseases such as cancer, cardiovascular disease, and neurodegeneration (Calderon-Montano et al., 2011). Moreover, many studies have demonstrated that kaempferol has a wide range of pharmacological properties, including antioxidant (Tatsimo et al., 2012), anti-inflammatory (Devi et al., 2015), antimicrobial (Tatsimo et al., 2012), anticancer (Yoshida et al., 2008; Chen and Chen, 2013), cardioprotective (Xu et al., 2006; Choi et al., 2015), antidiabetic (Zhang and Liu, 2011; Alkhalidy et al., 2018), and neuroprotective activities (Kim et al., 2010; Calderon-Montano et al., 2011). An *in vivo* study by Cheng et al. (2018) reported significant anti-neuroinflammatory effects of kaempferol through high-mobility group protein 1 release and decreasing the toll-like receptor-4/myeloid differentiation factor 88 which was induced by lipopolysaccharide in the brains of mice. The effective doses of kaempferol were 50, or 100 mg/kg for 7 days (Cheng et al., 2018). Kaempferol has also been shown to have an anti-apoptotic activity in Aβ-induced neuroblastoma cell lines which can be beneficial for the prevention and treatment of AD (Kim et al., 2019). Another study in the transgenic Drosophila model of AD indicated that administration of 10, 20, 30, and 40 μM of kaempferol for 30 days could delay memory loss, reduce oxidative stress and AChE activity, and therefore is a potential therapeutic agent for AD (Beg et al., 2018).

*Quercetin*, a flavonoid found in various foods including sorghum, possesses strong antioxidant activity (Zhang et al., 2011). It demonstrates anti-inflammatory activities, the mechanism of which is through inhibition of the NF-κB pathway (Comalada et al., 2005), anticancer activities through a variety of mechanisms (Xing et al., 2001), attenuation of high cholesterol (Lu et al., 2010), protection form viral infections (Davis et al., 2008; Gonzalez et al., 2009), reduced risk of diabetes (Vessal et al., 2003), and cardiovascular diseases (Kleemann et al., 2011). Several of these effects can in turn potentially reduce the risk of AD (Zaplatic et al., 2019). Pre-treatment of hippocampal cell cultures with quercetin considerably attenuates Aβ-induced cytotoxicity, protein oxidation, lipid peroxidation, and apoptosis (Ansari et al., 2009). Thus, quercetin might be protective against Aβ toxicity by regulating oxidative stress (Ansari et al., 2009). Moreover, quercetin deceases ROS which is a major contributor to AD (Zaplatic et al., 2019). It also decreases extracellular Aβ, AChE level, tau toxicity, and microgliosis (Sabogal-Guaqueta et al., 2015). Additionally, quercetin improved learning and memory function in aged 3xTg-AD mice determined through the elevated plus-maze test (Orhan et al., 2007; Sabogal-Guaqueta et al., 2015).

##### Dihydroflavonols

Dihydroflavonols also referred to as flavanonols, are a subgroup of flavonoids available in some plants including sorghum (Gujer et al., 1986). Common dihydroflavonols include taxifolin, dihydrokaempferol, and dihydromyricetin (Espargaro et al., 2017; Sunil and Xu, 2019). They have several reported potential health benefits including antiallergic and anti-inflammatory activities (Ayoub et al., 2018). Moreover, they show strong free radical scavenging activity and protect neuronal cells from oxidative damage *in vitro* (Gong et al., 2009). Dihydroflavonols also demonstrated the capacity to inhibit Aβ aggregation, a main pathological hallmark of AD (Espargaro et al., 2017).

*Taxifolin* is a flavonoid with strong anti-inflammatory and antioxidant activities (Topal et al., 2016; Wang et al., 2018). It is available from different natural sources such as onion, milk thistle, and sorghum (Sunil and Xu, 2019). It significantly attenuated Aβ-induced cognitive impairment and neuronal cell death which was measured through novel object recognition tasks and the spatial memory in a mice model of AD (Wang et al., 2018). The *in vivo* study of Saito et al. (2017) showed the capacity of taxifolin in the improvement of cognitive and cerebrovascular functions which was evaluated by the water maze test and monitoring the cerebral blood flow changes in the cerebral amyloid angiopathy model of mice.

###### Flavan-3-ols

Flavan-3-ols are a subgroup of flavonoids available in several natural sources including teas, apples, beer, wine, and cereals like sorghum (Yao et al., 2004; Rao et al., 2018). Flavan-3-ols have a variety of health beneficial effects including reducing metabolic syndrome risk (Yang et al., 2012), antioxidant activity (Castillo et al., 2000), anti-cancer characteristic (Lei et al., 2016) and neuroprotective potential (Bastianetto et al., 2006). Common flavan-3-ols are catechin, epigallocatechin, epicatechin, epicatechin 3-gallate, epigallocatechin 3-gallate (EGCG), and theaflavin (Yao et al., 2004).

*Catechins* are common in tea, cocoa, berries, and sorghum. They have potent antioxidant and anti-inflammatory activities as reported by several *in vitro* and *in vivo* studies (Higdon and Frei, 2003; Abd El-Aziz et al., 2012; Zanwar et al., 2014; Stohs and Bagchi, 2015). Apart from radical scavenging activity, catechins modulate mitochondrial functions, activate survival genes, and also fight against Aβ-induced cognitive deficit and neurotoxicity through their antioxidant activity (Heo and Lee, 2005; Ban et al., 2006; Haque et al., 2008). Therefore, catechins are receiving great attention as potential neuroprotective agents (Mandel and Youdim, 2004).

#### Non-flavonoids

##### Phenolic Acids

Phenolic acids are the simplest naturally available PP (Tsao, 2010). The natural sources of phenolic acids include fruits, vegetables, and cereals, especially sorghum (Klensporf-Pawlik and Aladedunye, 2017; Ratnavathi, 2019). The phenolic acids of sorghum are mostly benzoic or cinnamic acid derivatives (Calviello et al., 2007). Phenolic acids are reported to have strong antioxidant (Sroka and Cisowski, 2003) and anti-inflammatory activities (Kang et al., 2015) as well as other health benefits including neuroprotective activities (Saibabu et al., 2015).

*Cinnamic acid* is an aromatic carboxylic acid (see Figure 7) with many beneficial effects. Several studies have shown the anti-microbial, antioxidant (Sova, 2012; Guzman, 2014), anti-cancer (De et al., 2011; Su et al., 2015), anti-atherogenic (Lapeyre et al., 2005), anti-tuberculosis (De et al., 2012), and anti-fungal (Tawata et al., 2014) effects of cinnamic acid. Cinnamic acid treatment in a mouse model of AD significantly reduced the Aβ plaque formation and improved the cognitive function through PPARα activation to stimulate lysosomal biogenesis. Additionally, cinnamic acid treatment improved the memory and behavioral performance in the mouse model of AD (Chandra et al., 2019). Interestingly, cinnamic acid derivatives have been also reported to act as cholinesterase inhibitors thus may have therapeutic effects on AD through this mechanism (Lan et al., 2017; Chen et al., 2018).

*Ferulic acid (FA)* is the most abundant phenolic acid found in sorghum and is also suggested to have strong anti-inflammatory activity (Sosulski et al., 1982; Lempereur et al., 1997; Sgarbossa et al., 2015; Ratnavathi, 2019). Due to its chemical structure (Figure 7), FA possesses a strong free radical scavenging ability (Srinivasan et al., 2007). The antioxidant effect of FA has been shown to be effective against several chronic diseases such as cancer (Rocha et al., 2012), cardiovascular (Ardiansyah et al., 2008), diabetes (Jung et al., 2007), and cellular oxidative stress (Calabrese et al., 2008). The efficacy of FA has been investigated against several neurodegenerative pathologies, particularly in AD. According to the finding, it could inhibit fibril formation (Ono et al., 2005) and protect neurons against Aβ-induced oxidative stress and neurotoxicity *in vitro* (Sultana et al., 2005). Moreover, the *in vivo* study of Yan et al. (2001) demonstrated that long-term administration of FA induces resistance to Aβ toxicity in the brain likely through its antioxidant and anti-inflammatory. These results indicate that FA at a dosage of 5.3 mg/kg/day could be beneficial for the prevention and treatment of AD (Yan et al., 2013).

*Caffeic acid (CA)* is a hydroxycinnamic acid derivative which is commonly found in fruits, herbs, and grains, especially sorghum. It has strong antioxidant and anti-inflammatory activities (da Cunha et al., 2004; Gülçin, 2006; Priebe et al., 2014). According to the *in vivo* study of Kim et al. (2015), CA was administrated to a Aβ-injected mouse model of AD at an oral dose of 50 mg/kg/day for 2 weeks. The cognitive impairment was assessed by different behavioral tests. The result demonstrated the ability of CA to enhance memory and cognitive impairment through inhibition of lipid peroxidation and NO production (Kim et al., 2015).

*Sinapic acid (SA)* is another phenolic acid present in sorghum that is suggested to have anti-inflammatory and neuroprotective activity (Yun et al., 2008; Zare et al., 2015). The neuroprotective examination of SA (10 mg/kg/day for 7 days) in an Aβ-induced mouse model of AD showed a strong attenuation of glial cell activation and memory impairment in a passive avoidance task. Moreover, SA attenuated neuronal cell death and cognitive dysfunction through its antioxidant and anti-inflammatory activities (Lee et al., 2012).

##### Stilbenes

Stilbenes are important group of non-flavonoid PP produced by plants in response to major stress, especially, fungal infection, and UV radiation (Varoni et al., 2016). Stilbenes contain two benzene rings connected by ethanol or ethylene molecule (Yu et al., 2005). Stilbenes are present in some plants such as grapes, berries, and sorghum (Yu et al., 2005; Reinisalo et al., 2015). According to the literature, they are protective against cancer, cardiovascular disease and age-related disease through their antioxidant and anti-inflammatory activities (Reinisalo et al., 2015; Sirerol et al., 2016). More than 400 stilbenes are available in nature and the most well studied ones are resveratrol, pterostilbene, piceatannol, and pinosylvin (Sirerol et al., 2016).

*Resveratrol*, well known as a PP from grapes, is also found in sorghum grain. It is one of the most studied stilbenes for neuroprotection and AD prevention (Dal-Pan et al., 2017). Resveratrol is classified as a non-flavonoid PP. There are two isomers of this compound in plants, *trans*-resveratrol and *cis*-resveratrol, along with their glucosides, *trans*-piceid, and *cis*-piceid (Varoni et al., 2016). Clinical trials have shown the beneficial effects of resveratrol on neurological disorders, cardiovascular disease and diabetes biomarkers (Berman et al., 2017). Currently, resveratrol is considered as a nutraceutical due to its many therapeutic effects including the regulation of caloric restriction, anti-inflammatory, and antioxidant activities (Salehi et al., 2018; Banez et al., 2020). Numerous cell culture and animal studies of resveratrol have demonstrated its anti-inflammatory, antioxidant, anti-Aβ aggregation and anti-abnormal tau phosphorylation properties (Savaskan et al., 2003; Lagouge et al., 2006; Rege et al., 2015; Wang et al., 2016; He et al., 2017). Antioxidant and anti-inflammatory activity of resveratrol could increase the clearance of Aβ, and modulate oxidative stress, neuronal energy homeostasis, and apoptosis (Bastianetto et al., 2015). Resveratrol also assists synaptic plasticity and neuroprotective kinases activities (Bastianetto et al., 2015). It is also reported to provide its neuroprotective activity through the activation of SIRT1, an enzyme that deacetylates proteins related to cellular regulation (Lagouge et al., 2006).

##### Tannins

Tannins are a group of non-flavonoid PP with many biological activities specifically binding to precipitate proteins and other organic molecules (Hagerman and Butler, 1989). They protect plant from predation and also help plant growth (Ferrell and Richard, 2006). They are distributed in many plants including fruits, beverages and grains such as grape, coffee, tea, wine, cacao and sorghum (Lamy et al., 2016). Brown colored sorghum varieties are known to have a high antioxidant capacity due to their higher tannin content, which is not present in all genotypes of sorghum regardless of whether they are colored or not (Awika et al., 2004). Tannins have strong antioxidant and anti-inflammatory activities (Braidy et al., 2017b). They are reported to reduce hyperphosphorylation of tau proteins in *in vitro* study (Yao et al., 2013). Moreover, oral administration of tannins in a transgenic mouse model of cerebral amyloidosis demonstrated an improvement in object recognition and spatial reference memory (Mori et al., 2012) and also they showed to inhibit the β-secretase activity *in vitro* and therefore they have significant preventative potential against AD (Mori et al., 2012). Tannins also demonstrated a significant inhibitory effect against AChE and BChE (Türkan et al., 2019). Additionally, the study of Park et al. (2019) reported strong cognitive and memory enhancing activities of tannins in a rat model *via* avoidance and the water maze task.

## Conclusion

Currently available medication for AD is extremely limited in efficacy, therefore more studies should be conducted to discover new preventative and therapeutic agents. Recently, researchers have focused more on identifying treatments that can attenuate AD pathological hallmarks, rather than focusing on the treatments which only target the disease symptoms. Available symptomatic treatments such as AchEIs just attenuate symptoms temporarily by increasing the neurotransmitters in the brain without altering the disease progression path. For this purpose, studies on the effects of natural products such as polyphenolic antioxidants on AD pathological hallmarks are appearing in the scientific literature with increase regularity. One of the most highly concentrated food sources of antioxidant activity is sorghum grain which in colored gain varieties is due to high levels PP including 3-deoxyanthocyanidins, not found in any other common food.

To the best of our knowledge, there is no study on the effects of sorghum PP on AD pathology, therefore, the present review has illustrated the potential of sorghum PP as therapeutic agents against AD pathological hallmarks. This review has highlighted the unique chemistry and potential health beneficial properties of sorghum PP that can be leveraged to promote this under-utilized grain as a healthy food source.

As discussed throughout this review, numerous single PP have been studied and have demonstrated potential anti-AD effects in cellular and animal studies through a wide range of different mechanisms. However, a mixture of PP as found in an extract of sorghum grain could provide an additive or even synergistic multi-target therapeutic efficacy (Wang et al., 2014; Caruana et al., 2016; Andrade et al., 2019; Ayaz et al., 2019; Habtemariam, 2019).

Based on a variety of cell culture and animal model studies, sorghum PP have demonstrated several beneficial properties against some of the cellular pathways that contribute to AD pathogenesis. Among all the sorghum PP, caffeic acid, trans-resveratrol, quercetin, catechin, cinnamic acid, cyanidin, apigenin, and kaempferol have gained the most attention for their potential for AD prevention and treatment. However, the above-mentioned PP are not unique to sorghum. We hypothesise that the unique sorghum PP such as 3-DXA, and the complex mixtures of PP in sorghum grain extracts may collectively exert powerful synergistic effects on the inhibition of neurotoxic aggregation of Aβ and tau which initiate AD pathology.

Further studies to identify the specific mechanisms by which sorghum PP provide any neuroprotective activities are now necessary. One target mechanism is the antioxidant pathway in which the PP-rich extract of sorghum might reduce AD-associated oxidative stress. Both *in vitro* and *in vivo* animal model studies should be performed to gain as much evidence as possible before making recommendations for follow-on clinical trials. Moreover, anti-amyloidogenic, anti-tau/phospho tau, and anti-inflammatory mechanisms related to AD require further investigation. The new knowledge from these future studies may produce the high level of evidence require to confirm that the PP-rich extract from sorghum grain is a high efficacy preventative and therapeutic agent against AD.

## Author Contributions

NR, WMADB, EH, HS, SJ, SG, and RM substantially contributed to the conception and design of the article and interpreting the relevant literature. NR (PhD candidate), wrote the first draft of the manuscript. NR, WMADB, EH, HS, SJ, and RM revised it critically for important intellectual content. All authors contributed to the article and approved the submitted version.

## Conflict of Interest

SG is the owner of the CWEK Pty Ltd., WA, Australia. SJ is the Director of Ingredients by Design Pty Ltd. The authors declare that this study received partial funding from CWEK Pty Ltd. The funder had the following involvement in the study: Proofreading.

## Publisher’s Note

All claims expressed in this article are solely those of the authors and do not necessarily represent those of their affiliated organizations, or those of the publisher, the editors and the reviewers. Any product that may be evaluated in this article, or claim that may be made by its manufacturer, is not guaranteed or endorsed by the publisher.
